# Healthy Lifetime *(HL)*: An Internet-Based Behavioral Health Coaching Protocol for Older Adults

**DOI:** 10.3389/fdgth.2022.795827

**Published:** 2022-04-22

**Authors:** Marna Flaherty-Robb, Margaret Calarco, Susan Butterworth, Laura Struble, Karen Harden, Mary Franklin, Stacia Potempa, Candia Laughlin, Patricia Schmidt, Judith Policicchio, Olga Yakusheva, Deanna Isaman, Nancy Ambrose Gallagher, Philip Furspan, Kathleen Potempa

**Affiliations:** ^1^School of Nursing, University of Michigan, Ann Arbor, MI, United States; ^2^Q-Consult, LLC, St. Petersburg, FL, United States

**Keywords:** aging, chronic illness, health coaching, virtual, independent living

## Abstract

By 2060, the number of Americans 65 years and older will more than double, comprising nearly one-quarter of the population in the United States. While there are many advantages to living longer, a byproduct of aging is also a growing incidence of chronic illness and functional health limitations associated with a concurrent rise in chronic disease and disability that impair independent living in the community. We describe a personalized, behavioral health coaching protocol for early intervention that is delivered online to enhance a participant's independent functioning and to increase their self-care capacity with a goal to maintain independent living throughout aging. The electronic platform provides secure access to fillable surveys, health tracking, “just in time” communication with coaches and scheduling of two-way videos launched from the platform site. The 2-month protocol used two-way video conferencing which allowed high fidelity communication to sustain a complex behavioral intervention. Participants indicate high satisfaction with the intervention, the use of the platform, and the technology. While many health systems across the U.S. have ramped up virtual delivery of care in a proactive manner with now more than 70% of out-patient visits conducted through virtual delivery modes in some health systems, there remains much unevenness in this capability across the U.S. Our approach is to create a stable, interoperable, virtual outreach system for personalized professional health coaching that is complementary to medically oriented services that supports the health and functioning of participants as they age.

## Introduction

One of the most desired goals of people is to remain independent and at home as they age–an outcome made possible only with sustained health and optimal functioning (1, 2). By 2060, the number of Americans 65 years and older will more than double, comprising nearly one-quarter of the population in the United States (3). While there are many advantages to living longer, a byproduct of aging is also a growing incidence of chronic illness and functional health limitations that impair independent living in the community (4, 5).

Barriers to “aging in place” include emerging frailty, behavioral risk, memory impairment, lack of caregiver support, and home environments that do not accommodate limitations (6, 7). Typically, these barriers emerge later in the aging process making community-based living more challenging. Most services for the elderly focus on support for when maximal assistance is needed, such as in-home aides, frequent caregiving services, and expanded care coordination–all at high cost to the consumer and insurers (8, 9).

To address these challenges, we developed a protocol called Healthy Lifetime (***HL***) that *takes a different approach*. Unlike most recent and current programs for “aging in place”, (10) ***HL*** seeks to intervene early in the aging process when individuals have the best chance for longer term benefits of changing their health behavior, to stave off functional decline, and to minimize the onset or exacerbation of chronic conditions. And, for individuals who are experiencing any of the barriers described above, the ***HL*** program helps them achieve their highest level of functioning and self-care capacity, while integrating and collaborating with, *but not duplicating*, their medical services such as primary care, specialty care, and case management or medical social services. While the latter medically oriented services support clinical management, they do not focus on helping older adults maximize health and function by building self-care capacity, long term health behavior change, and the functional resilience necessary to sustain or regain independent living (11).

### HL Program Development

The purpose of program design was to create, and pilot test a standardized structure, dose, and coaching method that is accessible online provided by nurses trained in Motivational Interviewing (MI), Cognitive Behavioral (CB) and other evidence-based techniques. Prior metanalysis studies have shown health coaching employing MI and/or CB approaches to have varying methods and effects in producing health behavior change in adults (12). The methods of delivery most frequently used are in person, face-to-face or by telephone with a wide range of duration of each treatment episode (e.g., 10–90 min). The overall length of treatment over time ranges from a few sessions to multiple sessions over many months. Dorstyn et al. in their review found that there was considerable variation also in how motivational interviewing and other techniques were delivered (12). For example, MI was offered in conjunction with educational resources, referrals to cognitive rehabilitation or other CB techniques. A study included by Dorstyn et al. was 8 weeks duration with individual sessions lasting 10–90 min. Other types of individual or group programs directed at older adults with chronic conditions designed to improve self-care capacity and skills have typically lasted 6–8 weeks (12–14).

**Figure 1:**
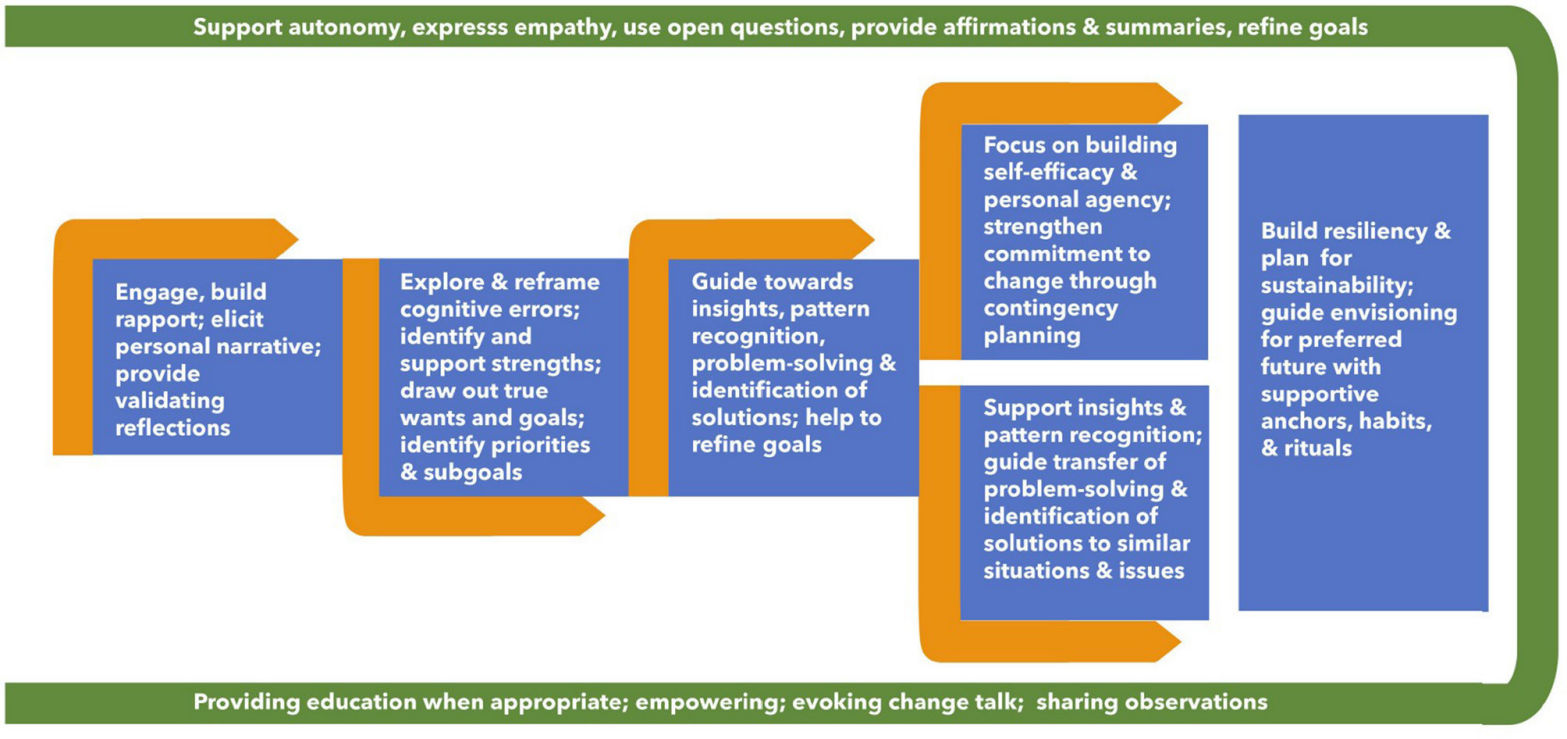
Process flow of health coaching weeks one through eight.

The ***HL*** program was structured to include weekly, 30-min coaching sessions and materials (Table 1) aligned with an evidence-based process of change (Figure 1) delivered over 8-weeks of intervention. The assessment survey is comprehensive, is in lay language, and includes items for demographic, socio-economic, clinical, and outcome measurement (Table 2). For example, the survey includes questions related to depression, sleep, and income, not included in outcome measures at this time, but were used as part of the assessment of the participant by the nurse coaches.

**Table 1:** Elements of the HL intervention.

**1. Provides a comprehensive assessment of the individual's health “eco-system” including the personal, environmental, social determinant, and financial impacts on health and health care and are re-evaluated over time;**
**2. Incorporates goals developed by the participant into an ongoing and evolving action plan with the guidance of the nurse;**
**3. Provides tools and methods for participants to understand enablers/barriers of the plan and gain sustainable skills in self-care management; and**
**4. Provides access to the nurse for teaching, coaching, and skill building in an easily navigable manner using video methods**.

**Table 2:** Personal health survey items.

**Demographic variables**
**Year of birth**
**Gender**
**Marital status**
**Race or ethnic origin**
**Highest level of education**
**Current work status**.
**Total household income**
**Social network and support variables**
**Spiritual beliefs**
**People to help me**
**Receives care from others**
**Level of engagement in social activity**
**Provides services to others**
**Has adequate transportation for activities**
**Income meets their needs**
**Health habits variables**
**Smoke tobacco?**
**Drink alcohol**
**Number of drinks per week**
**Level of exercise**
**Positive food choices**
**Negative food choices**
**Self-rated health**
**Health impact of chronic disease**
**Level of symptomatology**
**Confidence in ability to continue to manage symptomatology**
**Level of independence in activities [IADL items]**
**Self-efficacy in *ability to continue* essential life activities**
**Independent self-care agency/efficacy Scale**
**Medication taking self-care efficacy**
**Personal goal setting competency**
**Cost self-reported outcomes**

A workbook assists the participant in understanding their own health habits and in setting SMART (15) health goals and action planning that is intended for use initially and then throughout the program. The current ***HL*** protocol was pilot tested in several phases prior to its current entirely virtual delivery mode. Prior to the onset of the COVID pandemic, the surveys and in-person home assessment tools were pilot tested in a written, in-person format in 70 older adults ages 52–91. The surveys, home assessment tools, and health coaching were then converted to an electronic delivery format by providing an iPad with two-way video capability. In a pilot study with a sample of 14 older adult individuals (University of Michigan IRB approved), we found our participants were able to reliably complete online surveys and use the electronic system for two-way video communication. Additionally, feedback from the users indicated high satisfaction with this mode of delivery (Table 3).

**Table 3:** Satisfaction ratings with specific healthy lifetime program elements in program development phase.

**Program parts**	**Extremely satisfied** ***N* =**	**Very satisfied** ***N* =**	**Somewhat satisfied *N* =**	**Slightly satisfied** ***N* =**	**Not at all satisfied *N* =**	**N/A** ***N* =**
1x/week video coaching sessions	11	3				
Knowledge/Skills/Expertise of nurse	11	3				
2nd Nurse Home Visit (Creation of Action Plan)	11	2	1			
Quality of education/coaching	10	4				
The consent process	10	4				
The screening process	10	3				1
1st Nurse Home Visit (Clinical/Home assessments)	9	4	1			
The action plan itself	9	3	2			
Training from nurse on using iPad/Software	7	7				
The PHS Surveys	7	6	1			
iPad/Software program for video sessions	6	5	3			
iPad/Software program for daily logs	6	4	3	1		
IT consultants support	6	2	2			4
Daily audio recordings	4	3	5	1		1
Any educational materials given them by nurse	3	1	1	1		8
Strengthening plan workbook	2	5		2		5
Any referrals to community resources by nurse	2	2				10

The current ***HL*** protocol described in this manuscript is an 8-week health coaching model that includes motivational interviewing (MI) and cognitive behavioral (CB) and other evidence-based approaches (16) provided by trained nurse health coaches (see detailed description in Methods section). ***HL*** is designed to equip and empower older adults to achieve and maintain their health and function while also assisting them to manage the inevitable chronic conditions that come with aging. ***HL*** is a person-centered, personalized approach to maximize health and optimize functioning–the necessary requisite for individuals to successfully remain functionally independent in their preferred home setting, if possible, i.e., to age in place.

The ***HL*** program is delivered entirely on-line, delivered through an electronic, secure platform that provides easy password protected access, ability to complete and store surveys and health tracking information, and the ability to launch and store two-way video behavioral intervention [coaching] sessions from the platform. Both participants and the health coaches have access to all information stored on the platform (e.g., consent forms, completed surveys, and charting notes) and can provide “just in time” coaching through a “chat” function for participant/coach communication. The platform was modified to meet the purposes of our program and the needs of an aging population by increasing the font size and color contrasts used in the participant facing webpages and forms, and to support continuous “personalizing” to the individual participant's needs and progress by adding functions such as chats and tracking methods as they were needed and clearing distracting non-used functions from visibility.

## Protocol Study Methods

### Purpose

The primary purpose of the ***HL*** randomized intervention protocol, which is now in the 3-month follow-up phase, is to evaluate the benefits of the virtual 8-week program on selected health and functional outcomes, self-efficacy, and resiliency in a group of individuals over 50 years of age with one or more chronic conditions vs. a randomized control group of like individuals. The control participants are only given access to use the platform to fill out surveys and do not have access to other functions. Secondarily, we will evaluate: (1) the platform and protocol for ease of access and use by an older population, (2) the quality of delivery of a complex behavioral intervention through virtual means, and (3) the overall satisfaction of users with the program. A 3-month no-treatment follow-up phase will assess sustainability of participant engagement and benefits in ***HL*** and control group participants.

### Participants and Recruitment

The recruitment phase is now completed. The goal of this phase was to randomize a diverse group of ~120 individuals aged 50 and over who have one or more chronic conditions [e.g., hypertension, obesity, or functional decline] to either the ***HL*** intervention [58 individuals] or the control group [62 individuals]. Our sample size calculation was based on our regression model that accounts for using all three points in time in a single analysis. Using this model at a 5% alpha-level test, our proposed sample size of 120 participants has 80% power to detect an effect size of 0.07 which is considered a small-to-moderate effect. Also, our dropout rate thus far is <5%.

Inclusion and exclusion criteria are listed in Table 4. Participants in the ***HL*** and control groups were given a $150 honorarium for completing the 8-week phase and $50 upon completion of the 3-month follow-up phase. Recruitment occurred using several means to reach a diverse group of the older adult population of interest. Given the COVID pandemic, public display of flyers in community settings would not yield significant results because attendance at group events in indoor settings was significantly reduced during the recruitment phase of this study. First, we used the 2018 list of registered voters in Michigan [the most current list that included the name, address, and birth year of registrants by county]. In each of three waves of recruitment, we randomly selected 1,500 individuals from Michigan zip codes considered to have a greater density of people 50 years and older for a total of 4,500 mailings. *This approach to recruitment did not yield the expected number of responders*.

**Table 4:** Inclusion and exclusion recruitment criteria.

**Inclusion Criteria**	**Exclusion Criteria**
•50 years of age or older who have one or more chronic medical conditions (e.g., high blood pressure, diabetes, arthritis, obesity, etc.) which require management in some way (regular doctor checks, medication, etc.);	•Are acutely ill or have unstable health problems requiring medical work-up or follow-up clinic visits for monitoring more than every 3 months;
•Whose health is medically stable, that is, not currently undergoing either significant physical and/or mental health changes and not undergoing any type of Non-routine treatments/medical testing or have any surgeries scheduled in the next 6 months;	•Have had an ER visit related to his/her chronic condition in the prior 1 month; (an ER visit related to a *one-time, resolved issue* such as a bee sting or to have stitches for a household injury will not be cause for exclusion);
•Has not had an ER visit related to his/her chronic conditions in the prior 1 month (an ER visit related to a *one-time, resolved issue* such as a bee sting or to have stitches for a household injury will not be cause for exclusion);	•Are terminally ill;
•Can read, speak, and hear English; may use adaptive devices such as hearing aid and glasses;	•Have severe memory problems;
•Can recall personal information such as age, DOB, address, phone number, and health history questions without difficulty;	•Have severe hearing and/or visual deficits that are not functionally adapted with devices such as a hearing aid or eyeglasses;
•Reports having an established internet connection that is regularly used for video content [such as with Netflix, Amazon Prime, YouTube]; and	•Do not have an existing internet connection at the bandwidth needed to support the video platform [cannot access video streaming content]; and/or
•Can use their internet connection in a private space.	•Can use internet only in a public space (unable to ensure privacy).

Subsequently, we worked with the Michigan Department of Health and Human Services (MDHHS) to recruit Medicare and Medicare/Medicaid dual-eligible participants and with the Healthier Black Elders Center's registry in Detroit, Michigan, maintained by the Michigan Center for Urban African American Aging Research (MCUAAAR) (https://mcuaaar.org/cores/community-liaison-and-recruitment-core/participant-resource-pool/), to increase the diversity of our participant pool. Potential study volunteers were mailed the study flier and letter of invitation using regular postal mail and email blasts from the community centers' leaders. Additionally, we created a public research website that was posted to the University of Michigan's Health Research Group site for recruitment of participants. The use of websites and community center registry lists yielded the greatest number of study volunteers who met the target inclusion criteria, while also adding to the diversity of the participant pool.

### Study Design

Individuals who met inclusion criteria signed the informed consent to participate, and then completed the initial baseline surveys, were randomized to either the *HL* intervention or the control group. Randomization sequence was created using Excel 2007 (Microsoft, Redmond, WA, USA) with a 1:1 allocation using random block sizes of 2 and 4 by a blinded team member.

The ***HL*** group provided an 8-week active intervention that included: (1) an initial health and goal assessment survey completed by the participant on-line using the study electronic platform, (2) a health story narrative session, (3) a virtual home assessment to determine environment and safety, (4) an explanation of the surveys by the nurse coach, (5) a virtual session with the nurse coach to discuss overall health goals and action planning, and (6) six weekly 30-min personalized health coaching sessions with the trained nurse health coach. The specific elements of the intervention described in Table 2 were all conducted through a two-way, HIPPA compliant and secure video connection [Zoom Cloud Meetings]. The audio portions of the two-way video sessions were saved on a secure HIPPA protected platform [https://gethealthie.com], de-identified, and saved in the same location for later evaluation.

The control group had access only to the electronic platform for filling out the evaluation surveys during the 8-week and 3-month follow-up phase of the protocol. No other functions were available to the control participants. The control group participants filled out the survey items described in Table 2 and did not receive other intervention elements described in Table 1. We expect that both the ***HL*** and the control group members will derive some benefit from a focus on their health over the course of the study as may be prompted by answering the survey questions. The assessment survey asks health related questions that will likely prompt self-reflection of participants in both groups regarding their health status, health behaviors and lifestyle habits. The final set of questions of the assessment survey prompts identification of up to three top priority health related goals such as to lose weight, to eat healthier foods, exercise, etc. Our objective was to specifically determine the efficacy of the nurse health coaching 8-week treatment over and above an individual's routine personal attention to their health and healthy lifestyle.

### Measures

Several categories of measures were used for different purposes and are listed in Table 2. The specific questions are listed in the ***Personal Health Survey*** (PHS) provided in the Appendix. All scales are either public domain or used with permission as indicated. The PHS is online and accessible on the electronic platform at three measurement points of the protocol: at baseline, at 8 weeks and at 12 weeks. Participants are prompted to access the PHS at these three time points for completion. Baseline measures are visible to the participant only at the baseline measurement point. ***Demographic*** (PHS Q1–Q7) and the ***Social Network and Support*** (PHS Q8–Q16) information was measured only at baseline and created by the investigators to describe the sample and to better understand the participant's personal circumstance and needs for coaching purposes. All other questions were measured at the three time points of the protocol.

***Health Habits*** included questions about smoking, drinking, and use of marijuana (PHS Q23–Q26) which were “yes/no” responses and if “yes” additional items were added to describe frequency. ***Exercise/activity*** is a composite total score of five questions (PHS Q27–Q31) including stretching and aerobic activity items. Each question has a 5-point response choice of time/week of exercise. Test-retest reliability has been reported as ranging from 0.56 for the stretching item and 0.72 for the aerobic items in subjects with chronic health conditions (13). Food choices were PHS Q32–Q40 and included major food group recommendations of the 2015–2020 *Dietary Guidelines for Americans* (14). These items are used both for coaching purposes and as outcome measures. Outcomes are composite total score measures of ***Positive Food Choices*** (PHS Q32–Q34) as these represent groups recommended to be added for a healthier diet, and ***Negative Food Choices*** (PHS Q35, Q40) as these represent items recommended to be reduced for a healthier diet where higher scores represent better or worse choices, respectively (14). Psychometric properties of the two measures will be assessed in this study.

***Self-rated Health*** is measured in two different time dimensions: *Now* (PHS Q21), which is the standard 5-point Likert scale used in multiple studies (15–18) and *health in 3 years* (PHS Q22) which is a new 5-point Likert scale added by the investigators to measure projected sustainability of overall health. The specific prompting question of Q21 is taken from Self-Management Resource Center of Stanford University (SMRC) (19) with test-retest reliability of 0.92 in one study of 1,129 participants reported by Lorig et al. (13). Psychometric properties of the *health in 3 years* question will be assessed in this study.

***Health Impact of Chronic Disease*** is a composite total score measure of PHS Q41–Q44. Item responses are 5-point Likert scales where 0 is “Not at All” to 4 “Almost Totally” with higher total score values representing worse impact of chronic disease. This is the “Social/Role Activities Limitations Scale” of the SMRC with reported internal consistency reliability of 0.91 (Cronbach's alpha) and test-retest reliability of 0.68 (13). ***Level of*** **Symptomatology** is a composite total score measure of PHS Q45–Q50 of symptom frequency where higher score indicating more frequent or worse symptomatology. This is an adaptation of areas assessed by tools of the SMRC including pain, discomfort, shortness of breath, etc., selected for relevance to our participant population. Each item is a 6-point scale ranging from 0 “never” to 5 “always”. Psychometric properties of this adapted composite measure will be assessed in this study. ***Confidence in Ability to Continue to Manage Symptomatology*** is a composite total score of PHS Q65–Q70 with higher scores indicating higher confidence/efficacy. It is the SMRC “Self-Efficacy for Managing Chronic Disease 6-Item Scale,” which is a 11-point Likert scale ranging from 0 “Not at all Confident” to 10 “totally confident” with reported internal consistency reliability of 0.91 (Cronbach's alpha) (20).

***Level of Independent in Activities*** is a composite total score of PHS Q53–Q60 with higher scores meaning higher independence. It is the Instrumental Activities of Daily Living (IADL) checklist of the Senior Planning Services, Santa Barbara, California (used with permission). The 5-point Likert scale ranges from 1 “Cannot Do” to 5 “Can do independently”. Psychometric properties of this scale will be assessed in this study. ***Self-efficacy in Ability to Continue Essential Life Activities*** is a composite total score measure of PHS Q61–Q64 and is the “Social/Recreational Activities Scale” of the SMRC with reported internal consistency reliability of 0.82 (Cronbach's alpha) and test-retest reliability of 0.84 (13) ***Independent Self-Care Agency/Efficacy*** (21) is a composite total score measure of PHS Q71–Q73 with higher scores meaning higher self-agency/efficacy. Each item is a ten-point Likert scale ranging from 1 “Not at all Confident” to 10 “Totally Confident”. This measure was created by the investigators to assess self-care agency. Psychometric properties of the measure will be assessed and reported at study completion.

***Medication Taking Self-Care Agenc**y* is a composite total score measure of PHS Q74–Q85 and is the “Self-Efficacy for Appropriate Medication Use Scale (SEAMS)” adapted by Risser et al. (22). It is a 3-point scale ranging from 0 “Not at all Confident” to 2 “Very Confident”. Principal component factor analysis was used to evaluate validity of the SEAMS. Internal consistency reliability was 0.89 using Cronbach's alpha (22).

***Personal Goal Setting Competency*** (23, 24) relates to PHS Q90–Q97. Q90, Q93, Q96 are “open-ended” questions wherein participants fill in up to three priority goals that they are working on at each of the three time points of study measurement–baseline, at 8 weeks and at 12 weeks. Goals as listed will be placed into “like” categories for all participants to describe priority goals of the sample over time. PHS Q91, Q94, Q97 rate the importance of each goal listed by a participant (up to three goals). *Average goal importance* is the sum of the importance score for each goal provided divided by the number of scores (e.g., Q91+Q94+Q97/3). The importance question is a 11-point Likert scale ranging from 0 “Not Important at All Now” to 10 “Highest Importance Now”. *Average confidence in achieving the goal score* is the sum of the confidence score for each goal divided by the number of scores (e.g., Q92+Q95+Q98/3). The confidence question is a 11-point Likert scale ranging from 0 “Not at all Confident” to 10 “Completely Confident”. ***Personal Goal Setting Competency*** is the sum of the score of *average goal importance* and the *average confidence* in achieving goals. It is expected that personal goal setting competency improves with nurse health coaching over time. Psychometric properties of the goal importance and confidence to achieve the goal scales will be evaluated in this study.

***Cost self-reported Outcomes*** include unplanned medical clinic visits (PHS Q17) where higher scores are more visits; Emergency room visits (ER) (PHS Q18) where higher scores mean more ER visits; and overnight stays in the hospital (PHS Q19) where higher scores mean more hospital stays. The questions were from SMRC with reported test- retest reliability of 0.76 for clinic visits, 0.94 for ER visits, and 0.97 for hospital stays (13). The cost questions were adapted for each measurement time point of the study to reflect a time frame of 1 month prior to the outcome measurement.

### Analysis

Intervention and control participants remain in the study for a 3-month (90-day) no treatment follow up period after the 8-week active intervention or control treatment to determine sustainability of engagement and if any benefits are achieved after the 8-week period. Quantitative self-reported outcome measures for all intervention and control participants are described in Table 2 and are taken at three time points–at the beginning of the study before randomization, at the end of the 8-week active intervention or control period, and after the 3-month no treatment follow-up phase. Participants fill in the responses themselves using the electronic platform on the fillable forms for the survey items. ***HL*** program staff check only to make sure forms are completed.

Quantitative analysis will include descriptive statistics means and standard deviations for continuous variables and frequencies for categorical variables. To assess the impact of the HL intervention on participant outcomes, we will use regression modeling with fixed-effect repeated measures to account for the longitudinal data collection at baseline, 8 weeks, and 12 weeks. For dichotomous outcomes, we will use logistic regression, and for continuous outcomes, we will use linear models with transformations as needed. The outcome variables are listed in Table 2. Independent covariates will include race, sex, whether the participant had a partner, education, and income. The primary variable of interest is the interaction between group and time. This interaction indicates a change in trajectory over time between the HL group and the control group.

To prevent over fitting, we will use a stepwise selection technique at an alpha-level of 0.05. This approach will allow us to identify a parsimonious set of variables that are independently associated with the outcome variables. To test the fit of the model, we will examine residual and quantile plots. Trajectories over time and interaction plots will be reported.

Additionally, we will conduct a qualitative analysis of the audio recordings of the ***HL*** intervention group to assess how the participants responded to the coaching in terms of process and qualitative outcomes expressed by the participants. Random sampling of 20-min audio segments will be pulled from each participant's audio files across the 8-week treatment period such that there is one segment from each of the treatment phases: the first 3 weeks, the middle 3 weeks, and the last 2 weeks of treatment. Constructs and related indicators to be coded are listed in Table 5. The qualitative assessment coding will be done by experienced coders who are trained in conducting analysis of coaching sessions [Q-consulthealthcare.com]. Inter-rater reliability of coders will be established by double coding a random sample of 10% of the coded sessions of each of 4 coders. We will use the online system from www.random.org to randomly select 10% of sessions from each coder.

**Table 5:** Qualitative outcome measures.

**Construct**	**Indicators**	**References**
Insight & Pattern Recognition	•Learning something about yourself (strength or weakness) •Expressing discovery of true sticking point or new goals and desired behaviors •Re-ordering of priorities (being honest with oneself) •Identifying pattern through tracking/journaling •Pursuing intentional thoughts and behaviors and associating them with pleasurable feelings or to health •Recognizing distractions that interferes with execution of intended goal or activity	(25–27)
Self-Efficacy & Personal Agency	Qualitative Coding Matrix Descriptors: •Expressing confidence for meeting goal Expressing confidence for overcoming barrier •Expressing belief that efforts make a difference •Expressing belief sense that one controls risk/destiny (locus of control) •Expressing appreciation of/sense of pride in specific progress actions or accomplishments	(28–30)
Building toward Sustainability	Qualitative Coding Matrix Descriptors: •Forming habit •Forming ritual •Expressing pleasure or benefit from new self-management activities •Forecasting desires and activities in future (“down the road”) •Connecting a desired prioritized activity to an existing habit or pleasurable ritual (“habit stacking”) •Developing plan/Imagining self in future for specific structured activity with time, place, circumstances (envisioning) (contingency planning) •Identifying/utilizing social support to enhance support for desired goal/activity	(27, 31–34)
Resiliency	Qualitative Coding Matrix Descriptors: •Expressing a reaction to perceived positive event/success •Expressing a reaction to perceived negative event/failure/risk Problem-solving for barrier •Engaging in trial and error •Re-committing after trial and error •Seeking guidance/resources from others/community •Identifying/choosing sequence of small steps toward goal	(35–38)
Change Talk		(39–41)

### Electronic Platform Functionality

The platform for video and audio is Healthie (https://gethealthie.com/). All recordings are initiated in HIPPA-compliant Healthie, downloaded to the nurse coach's secure hard drive, and uploaded to the University of Michigan's secure Dropbox cloud. All participant electronic survey data is stored on the Healthie platform in a separate, secure file for an individual linked only to their unique participant study ID. The audio data is voice distorted using Adobe Audition software and accessed via encrypted connection or from a local encrypted and secured workstation.

The platform provides for the following functionality. Surveys for evaluation are accessed and “fillable” on the platform by participants using standard Windows document software, are automatically stored upon completion, and able to be retrieved by participants and the study team. Participants have tracking functions accessible to them for inputting their personal information such as daily weights, blood pressures measurements, food choices at a frequency of their choice. A “chat” function is available for the participant and nurse coach to communicate through text messaging on an *ad hoc* basis. The two-way video sessions are scheduled using the scheduling system on the platform and are launched from the platform, both of which are secure and HIPPA compliant. Emails to/from participants can be launched from the platform. Importantly, the individual participants' ***HL*** record created during the program is interoperable with other electronic medical records. As well, HIPPA protected communication with other healthcare providers and exchange of information such as laboratory results from medical practitioners and health progress from the ***HL*** platform can occur. Even though we chose not to use this interoperability function in this ***HL*** protocol, this functionality is important in the long-term integration of health and medical services.

### Personalized Nurse Coaching Intervention

The registered nurse coaches have a minimum of a bachelor's degrees in Nursing, are experienced (10 or more years), and were provided 24 h of additional training in nurse health coaching strategies and methods. In addition, nurse senior coaches received regular mentoring and feedback sessions throughout the program by nurse coach and motivational interviewing (MI) experts. A weekly review of individual participant “cases” by the nurse coach group with experienced experts provided additional oversight of the coaching process to sustain fidelity to the coaching methodology.

**Figure 2:**
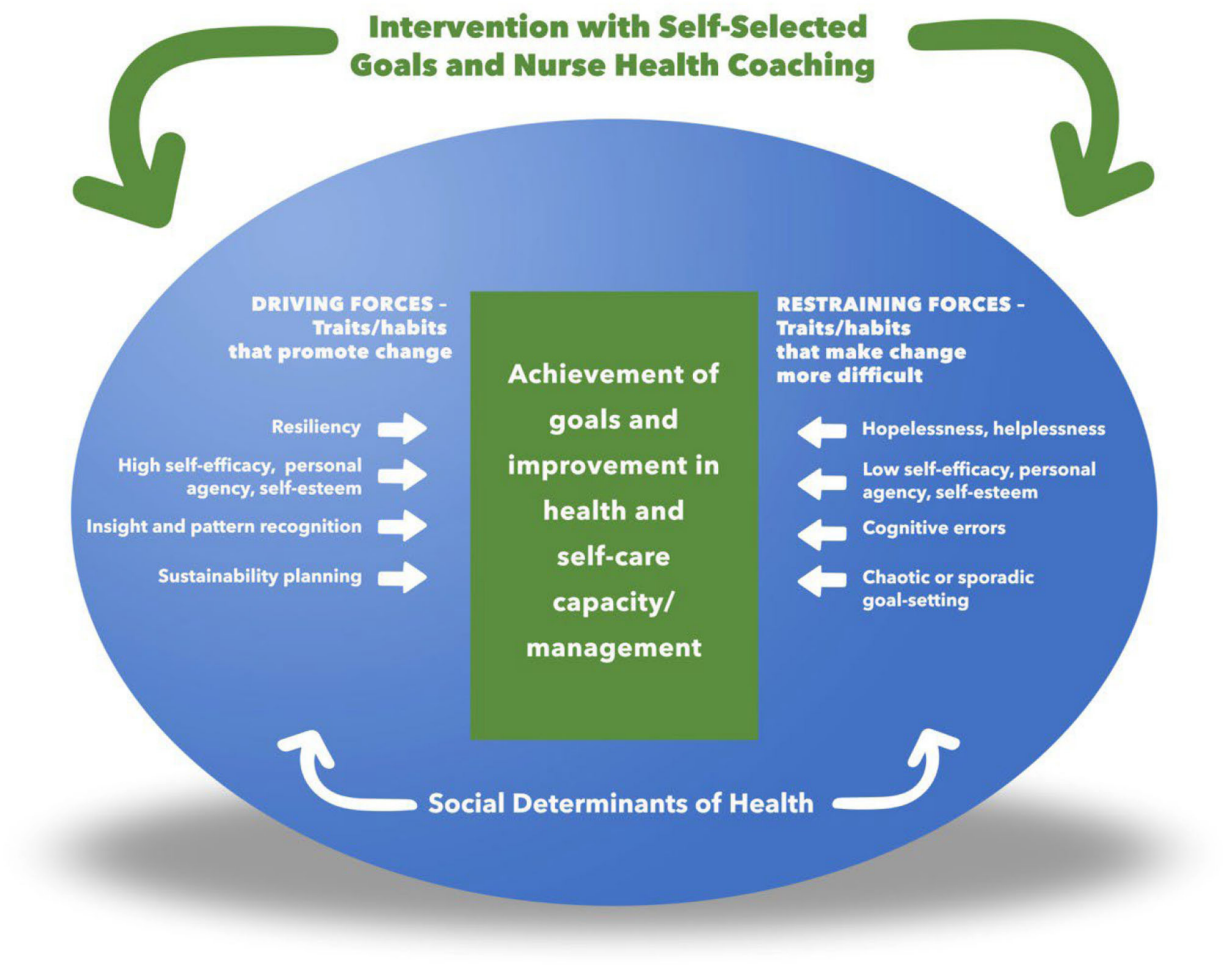
A Model of personalized framework for nurse health coaching [adapted from the concepts of Lewin Field Theory (45)].

The coaching strategies employed focused on person-centered engagement, understanding and reflecting story, client empowerment and independence, cognitive-behavioral as well as narrative coaching approaches, with the overall communication approach based on MI communication techniques (42). This blend and balance of coaching strategies was focused on building self-care capacity, improved functioning, and reflective and problem-solving skills aimed at improving overall health literacy, resiliency, self-efficacy, and quality of life (42–44). The strategies were personalized to address both driving and inhibiting forces to behavior change (Figure 2) as these were revealed during the course of each individual's coaching experience. We also considered several dimensions of the social determinants (46) of health in the understanding and application of nuanced coaching strategies.

The complex behavioral intervention we employed requires attention to the participants' physical and verbal responses that are dependent on high fidelity video and audio reception. During the intervention, the quality of the reception was monitored and reported by the nurse coaches. An experienced information technologist familiar with the platform was available during the scheduled hours of coaching and provided “just in time assistance” when needed. This technician worked closely with a senior nurse information officer familiar with best usability characteristics. There were occasional times (on average 2x a month) when the video session required assistance from the technologist, usually to assist the participant with their connection. There were very few times when the video session was not able to be conducted even with the technologist's intervention. In these rare events, the session was rescheduled. We have found that as the participants gained experience with use of the video launch and sessions, there was mutual realization of the time of day with lower internet use in their area and schedules were adjusted to accommodate.

## Discussion, Ethical Considerations, and Dissemination

Population health has been defined as “the health outcomes of a group of individuals, including the distribution of such outcomes within the group” (47). It focuses on the health of entire populations of specific definition. Typically, consideration of a population's health includes addressing in depth understanding of population needs in their own experience, health outcomes, patterns of health determinants including social determinants of health, and the policies and available interventions accessible to the population (47). The rapid escalation of chronic diseases, functional limitations, and disability with age in the U.S. and other aging populations (48) is associated with a high incidence of conditions such as obesity, smoking, and inactivity associated with lifestyle choices (49–51). This circumstance indicates the need to dramatically expand the attention to promoting health, not just managing disease (52).

Yet, most health promotion programs conducted in health care settings focus on early detection of disease through medical surveillance of disease precursors such as laboratory measures of blood sugar, hemoglobin a1c, serum cholesterol, blood pressure, body weight, etc. While important, this attention on early detection alone does little to stave off the emergence of chronic diseases in the long term (53). While the aging process will inevitably bring health decline, the best possible outcome is for this decline to be delayed as long as feasible (54). The gain in “quality life years” (55) from improving health behaviors as well as health decisions and choices will potentially improve overall quality of life and reduce the cost of care in the later years of life (56).

The variability in health coaching approaches for adults makes it difficult to determine the best practice for such programs in clinical settings (12, 13). Additionally, there is currently a paucity of health promotion programs that address these issues that are available, accessible, personalized, and effective for the older adult population (57). While health coaching is gaining increased attention in the health care setting and by health care insurers, adequate evidence-based guidance on program design hinders broad-based dissemination and predictable benefit. The ***HL*** protocol is a standardized structure, dose, and coaching method that is accessible online provided by nurses trained in Motivational Interviewing (MI), Cognitive Behavioral (CB) and other evidence-based techniques designed to provide a model for program design and dissemination.

The ***HL*** protocol is being tested for efficacy in improving health behavior, health decision making and problem solving, and independent self-care agency for taking control of health choices for the long-term. The electronic format provides for access and scalability beyond the geographic limits of most health system service delivery models (58, 59). Accessibility is an important ethical consideration especially for older adults who may not be fully able to travel to/from in person programs or who may live in rural areas where programs are not immediately available. The State of Michigan has invested in extensive broadband penetration throughout the state such that on-line programs are accessible even in remote areas where in person health promotion and other supplemental health care programs are less available (60).

Further, while the COVID pandemic has challenged health systems to accelerate the use of electronic formats to deliver health care, the expansion has predominantly addressed primary care and routine medical surveillance follow-up (61). There are numerous reports about the methods and lack of veracity of technology for health follow-up especially those quickly ramped up to meet the needs of people during the pandemic (62–64). While many health systems across the U.S. have ramped up virtual delivery of care in a proactive manner with now in excess of 70% of out-patient visits conducted through virtual delivery modes in some health systems, (65) there remains much unevenness in this capability across the U.S. Our approach is to create a stable, interoperable, virtual outreach system for personalized professional health coaching that is complementary to medically oriented services that support the health and functioning of participants.

An essential feature of the electronic platform we use is its interoperability with other electronic systems used to store medical records. The ability to integrate health promotion services with existing health systems is of significant interest and importance. The capability of the ***HL*** program platform to provide stable, accessible, and secure collection of survey and other health information, launch and record video interactive sessions, store libraries of auxiliary health information, launch asynchronous and synchronous chat rooms, schedule appointments, and other fully integrated services will strengthen the use of telehealth and electronic service delivery as a major modality–not just used in times of pandemic crisis. In addition, no other designed electronic record is focusing on the recording of decision making, problem solving and trial and error learning in self-care, to assist clients to select and mature successful strategies through time and circumstance. Social determinant indexing is now common, but we do not now routinely address or record self-care capacity skill building progress–which when combined, will provide the understanding of the interaction of social determinants and capacity building methods and the opportunity to maximally improve functional outcomes in vulnerable populations.

Ultimately, a healthier population is fostered by scalable health promotion methods not bound to a particular geography or a particular health system's reach (59). The opportunity that such capability provides is to accelerate the access to health promotion for dissemination to targeted and vulnerable populations to achieve better health outcomes.

## Data Availability Statement

The raw data supporting the conclusions of this article will be made available by the authors, without unduereservation.

## Ethics Statement

The studies involving human participants were reviewed and approved by University of Michigan Health Sciences and Behavioral Sciences Institutional Review Board (IRB-HSBS). The patients/participants provided their written informed consent to participate in this study.

## Author Contributions

All authors listed have made a substantial, direct, and intellectual contribution to the work and approved it for publication.

## Funding

This study is funded by the State of Michigan Health and Human Services Department AWD019289-SUB004 and the University of Michigan School of Nursing.

## Conflict of Interest

SB is the Principal of Q-Consult and is employed by the University of Michigan as a behaviorist who assisted with training the nurse coaches in motivational interviewing techniques. The remaining authors declare that the research was conducted in the absence of any commercial or financial relationships that could be construed as a potential conflict of interest.

## Publisher's Note

All claims expressed in this article are solely those of the authors and do not necessarily represent those of their affiliated organizations, or those of the publisher, the editors and the reviewers. Any product that may be evaluated in this article, or claim that may be made by its manufacturer, is not guaranteed or endorsed by the publisher.
